# Emerging Perspectives on Platelet-Activating Factor in Relation to Magnesium Levels at the Cellular, Tissue, and Systemic Levels in Disease States

**DOI:** 10.3390/cells15050419

**Published:** 2026-02-27

**Authors:** Amanda Kaine, Anthony Gariolo, Andreas Karaolis, Luv Kataria, Ezaan Khan, Dhruv Mayank Patel, Sidhartha D. Ray, Nilank Shah

**Affiliations:** 1 Touro College of Osteopathic Medicine, 60 Prospect Ave., Middletown, NY 10940, USA; akaine@student.touro.edu (A.K.); agariolo@student.touro.edu (A.G.); akaraolis@student.touro.edu (A.K.); lkataria@student.touro.edu (L.K.); ekhan7@student.touro.edu (E.K.); dpatel90@student.touro.edu (D.M.P.); 2 Touro College of Pharmacy, Pharmaceutical and Biomedical Sciences, 3 Times Square, Room 856, New York, NY 10036, USA

**Keywords:** magnesium, PAF, calcium, hypomagnesemia, hemorrhage, insomnia, hypoxemia

## Abstract

**Highlights:**

The objectives of this review paper are to provide further context and insight into the interrelationship of PAF and magnesium within the clinical context.

**What are the main findings?**

- A main finding of this paper was the antagonistic nature of PAF and magnesium. Of note is that hypomagnesemia correlated with increased PAF activity and increased platelet activation.
- Renal disease, such as chronic kidney, disease provides measurable and statistical support when evaluating the value of using PAF as a biomarker for prognostic disease progression.

**What are the implications of the main findings?**

- The implication for this finding is supported through our evaluation of the inflammatory markers in endothelial cells, where reduced magnesium aids in suppressing the calcium overload stimulated via PAF =-mediated inflammatory processes.
- The implication of this finding is supported by the description of increased urinary and plasma PAF level findings, as well as increased plasma platelet-aggravating factor acetylhydrolase (PAF-AH) activity in patients with primary glomerulonephritis.

**Abstract:**

Magnesium is an essential micronutrient that exerts fundamental roles at both the cellular and tissue levels, with broad therapeutic and preventive implications across a range of pathological conditions. Accumulating clinical and experimental evidence indicates that optimal magnesium homeostasis modulates key pathophysiological processes and serves as both a biological and prognostic marker in disorders such as stroke, myocardial infarction, type 2 diabetes mellitus, and renal failure. These disease states commonly originate from two major etiological determinants—hypertension and atherosclerosis—which share a unifying pro-inflammatory mediator: platelet-activating factor (PAF). PAF plays a central role in vascular inflammation by promoting platelet aggregation, macrophage infiltration, leukocyte adhesion, and vasomotor dysregulation. Importantly, magnesium demonstrates an inverse association with both platelet aggregation and PAF activity, underscoring its protective capacity in mitigating vascular inflammation and preserving endothelial function. The objective of this updated literature review is to elucidate the antagonistic interplay between magnesium and PAF, with a focus on its physiological and therapeutic significance across multiple organ systems. While emerging data support a modulatory role of magnesium in PAF-mediated inflammatory pathways, current evidence remains limited. Therefore, further mechanistic, pharmacological, and clinical investigations are warranted to clarify the multifaceted role of magnesium in attenuating PAF-driven disease processes.

## 1. Introduction

Magnesium deficiency, or hypomagnesemia, frequently arises under conditions of chronic stress, malnutrition, cardiovascular dysfunction, and other states characterized by endothelial vulnerability. Beyond its autonomous physiological functions, magnesium interacts with a range of biochemical mediators to regulate vascular tone, modulate inflammatory responses, and maintain endothelial integrity. Among these interactions, magnesium’s modulatory effect on platelet-activating factor (PAF) is of particular interest. PAF, a potent phospholipid-derived mediator, triggers macrophage activation and intracellular calcium signaling, initiating downstream inflammatory cascades. Magnesium counterbalances these effects by inhibiting calcium influx into vascular smooth muscle cells, thereby promoting vasodilation and reducing vascular resistance. Through this calcium-antagonistic mechanism, magnesium mitigates hypertension, limits cardiac hypercontractility, and supports vascular homeostasis.

This review examines the complex interplay between magnesium and PAF and its clinical implications across diverse disease states. Special attention is given to magnesium’s role in antagonizing PAF-mediated biochemical and inflammatory pathways implicated in cardiovascular, renal, neurological, and metabolic disorders. By integrating emerging molecular and clinical evidence, this review underscores magnesium’s potential as a modulator of endothelial function and systemic inflammation, highlighting its relevance in both preventive and therapeutic contexts.

## 2. Chemical Structure

PAF, a potent phospholipid mediator, triggers platelet activation, cell signaling, and plays a role in various physiological processes. This structure is known as PAF acether or AGEPC (acetyl-glyceryl-ether-phosphorylcholine). This molecule is an ether-linked glycerophospholipid characterized by an alkyl ether bond or an acetyl group and a phosphocholine head group [1].

**Figure 1:**
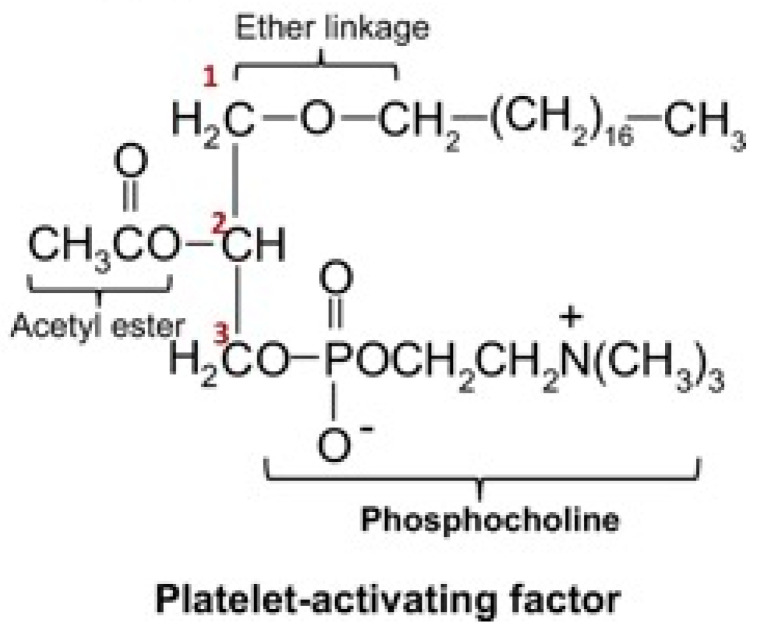
Structure of platelet-activating factor. Structural features of PAF include: (1) an ether bond at the sn-1 position. (2) Acetic acid esterified to glycerol at sn-2. (3) Phosphocholine head group at sn-3. By Damiani, E. & Ullrich, S.E.

This unique structural configuration underlies PAF’s highly multifaceted biological capabilities, with its ability to interact with other membrane-based molecules through its PAF receptor and signaling pathways, as seen in Figure 1 [1]. These signaling pathways can induce cytokine release, enzymatic regulation, inflammatory processes, and amplify various vascular responses.

## 3. History

Platelet-activating factor (PAF) was initially discovered in the early 1970s and characterized as a potent phospholipid mediator that induces platelet aggregation. In the early stages of PAF research it was found to have implications during anaphylactic and immune-mediated events, specifically through leukocyte-dependent platelet activation and histamine release [2,3].

By the late 1980s and early 1990s further research had established PAF as a pleiotropic inflammatory mediator synthesized by cytokines, hypoxia, and oxidative stress [4,5]. Further PAF research identified it signaling cascades through G-protein coupled receptors that activate phospholipase C (PLC), inositol triphosphate (IP3), diacylglycerol (DAG), and intracellular calcium [6]. These findings highlighted the importance of PAF as a potent signaling molecule linked to inflammation, thrombosis, vascular tone, and cellular adhesion.

The discovery of platelet-activating factor acetyl hydrolase (PAF-AH) was later discovered as the primary enzyme responsible for PAF degradation [7], emphasizing the importance of its physiological regulation in inflammatory disease progression [8]. These findings not only established PAF as an inflammatory mediator but also as a regulator whose imbalance can cause pathological disturbances.

In summary, the history of PAF began as a platelet-activating factor to its current implications as a prominent interconnected network of inflammatory, thrombotic, and calcium-dependent channels, thus highlighting its importance in disease progression [4,6,9]. These concepts prompt further investigation into modulators of PAF such as magnesium, which may suppress PAF inflammatory and endothelial dysfunction systemically.

## 4. Calcium and Magnesium Relationship

Magnesium exhibits both synergetic and opposing interactions with a key electrolyte, such as calcium. The divergent physiological roles of calcium and magnesium are particularly evident in the cardiovascular system, where both ions are critical for maintaining cardiovascular homeostasis. Calcium is a primary second messenger that triggers myocardial and vascular smooth muscle cell contraction and promotes vasoconstriction. In contrast, magnesium functions as a physiological calcium antagonist. It modulates cardiovascular function by competing with binding sites on cell membranes and regulatory proteins, thereby inhibiting calcium influx into smooth vascular muscle cells. This action promotes endothelial-dependent vasodilation and muscle relaxation, counterbalancing the contractile effects of calcium. Thus, this calcium channel blocker mimicry, or antagonist effect, can be beneficial in improving vascular relaxation and confer cardioprotective effects for those at risk of hypertension and cardiovascular disease.

To further explore this relationship, a calcium-to-magnesium ratio has been studied to correlate the risk of cardiovascular and inflammatory diseases. A study reported that elevated calcium levels are associated with systemic inflammation and higher magnesium concentration, which shows an inverse relationship with IL-6 expression [10]. Such findings indicate the importance of maintaining an optimal calcium-to-magnesium balance to prevent platelet aggregation and suppress inflammatory signaling.

**Figure 2:**
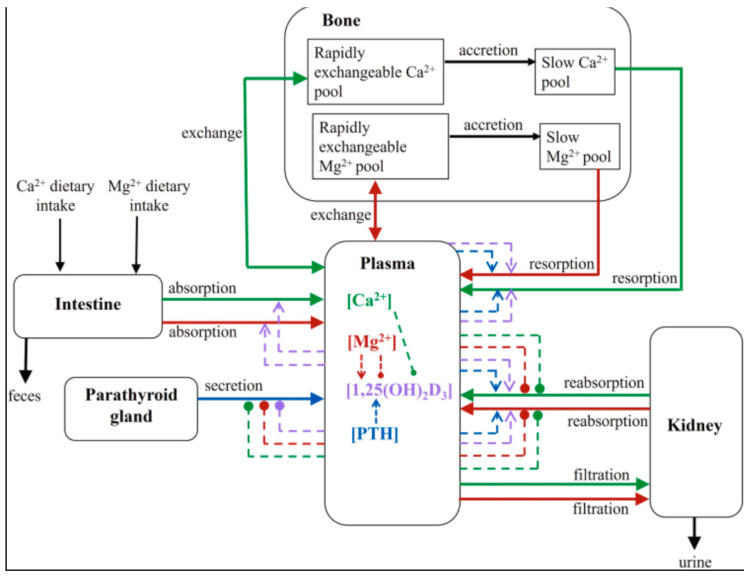
Schematics of the Mg^2+^ homeostasis model. The model consists of five compartments: plasma, intestine, kidney, parathyroid gland, and bone. Arrows with triangular arrowheads indicate activation, while those with circular arrowheads indicate inhibition. All arrows are color-coded: green, Ca^2+^; red, Mg^2+^; blue, parathyroid hormone (PTH), a calcium, vitamin D, and phosphate regulator, which is a peptide hormone produced by four small parathyroid glands in the neck. Also depicted is a magnesium-regulating hormone secreted by the parathyroid glands (1,25-dihydroxyvitamin D_3_ [1,25(OH)_2_D_3_]) which is depicted as a mauve arrow. 1,25-dihydroxyvitamin D_3_ is a biologically active form of vitamin D. It is also known as Calcitriol, which is involved in intestinal mineral absorption, calcium regulation, functions as an immunomodulator, and is heavily involved in promoting bone metabolism. [Original pathway contributed by Nilank Shah, MD, and collaborators].

Clinical studies have explored the observable dietary role of magnesium and its impact when deficient, leading to marked hypomagnesemia, accompanied by mild hypocalcemia. Severe pathological states of hypomagnesemia, indicating low serum magnesium levels, can lead to a paradoxical block of secretion by parathyroid hormone [11]. In addition, hypomagnesemia causes resistance to PTH at target tissues, some sites being bone, intestines, or the kidneys, as shown in Figure 2, which can further exacerbate hypocalcemic conditions. Consequently, calcium levels cannot normalize until magnesium deficiency is corrected, and thus, this reciprocal relationship can promote the states of various hormonal levels, such as magnesium and calcium levels in the body. The pathway of these interdependent states of magnesium, calcium, and parathyroid hormone (PTH) as illustrated in Figure 2, clearly depicts magnesium’s role across five physiologic compartments and its associated hormonal feedback-dependent mechanisms [11].

This relationship is illustrated in Figure 2, demonstrating the relationship of magnesium on five compartments, and its involvement of hormonal feedback mechanisms [11].

Based on limited evidence, studies indicate that PTH influences blood levels of platelet-activating factor (PAF), particularly in patients with end-stage renal disease [12]. Research suggests that high levels of parathyroid hormone are associated with increased PAF levels in hemodialysis patients, suggesting a role in cardiovascular and inflammatory complications. Elevated PTH levels, commonly found in CKD and hyperparathyroidism, are linked to an increased risk of atrial fibrillation (AF) precipitating inflammation triggered by PAF which serves as a potent phospholipid mediator of inflammation, cardiovascular dysfunction, and platelet aggregation. Research on hemodialysis patients has specifically investigated the link between PTH and PAF, establishing a connection between higher PTH and increased PAF levels, suggesting that PTH may likely promote inflammation via this pathway.

## 5. Calcium and PAF Relationship

**Figure 3:**
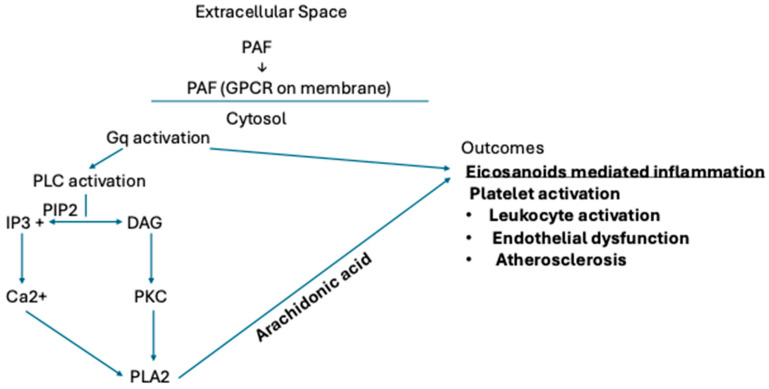
Image modified by Kaine, A. Calcium and IP3 DAG activation pathway and associated clinical outcomes.

Activation of macrophages has been shown to elevate intracellular calcium concentration under PAF regulation. Such mediators include proinflammatory cytokines, such as thrombin, and vasoactive mediators [6]. This cascade of events is initiated by the signaling of PAF binding to a specific G-Protein-coupled phospholipid receptor, known as PAF receptor (PAFR) [6]. Upon receptor engagement, downstream signaling promotes generation of IP3, which mobilizes and subsequently releases intracellular calcium stores, as illustrated in Figure 3 [6]. Elevated intracellular calcium likely amplifies inflammatory signaling, macrophage adhesion, and contributes to plaque formation, considered a hallmark of atherosclerosis.

Elevated intracellular calcium acts as a critical second messenger that amplifies inflammatory signaling within macrophages, enhancing cellular adhesion, cytokine release, and promoting foam cell formation. The macrophage activation pathway, which involves upregulation of PAF and is illustrated in Figure 3, has been linked with increased oxidative stress and plaque formation. PAF, found activating these platelets, induces aggregation and endothelial disruption [8]. This sustained PAF signaling pathway also requires phospholipase C (PLC)-mediated activation perpetuating the calcium release further and continuing to reinforce the pro-inflammatory process. In totality, when PAF is active, the calcium-dependent signaling cascade can further drive the progression of atherosclerosis, given the endothelial instability and amplified vascular remodeling effects that high intracellular calcium elicits.

Conversely, magnesium can serve to stabilize this calcium overload through its antagonistic effects on calcium channels. By limiting calcium influx, magnesium attenuates platelet activation, reduces platelet excitability, and dampens the pro-inflammatory signaling pathways upregulated by calcium and PAF. This downregulation of calcium, mediated by magnesium, further elucidates the important role of magnesium for therapeutic purposes. Due to the protective role of magnesium, magnesium can be used for therapeutic supplementation for many disease states. Magnesium has been studied within the cardiovascular system as both an agent of therapeutic supplementation and as a prognostic marker for inflammation and ischemic heart disease.

## 6. Ischemic Heart Disease/Myocardial Infarction and Magnesium Role/PAF

Magnesium levels have long been associated with numerous cardiovascular diseases, including atrial arrhythmias, strokes, and coronary heart disease. In the context of ischemic heart disease and myocardial infarction, magnesium has been found to reduce calcium overload in the myocardium, thereby improving cardiac function [13]. Postmortem analyses reported decreased myocardial magnesium in ischemic heart disease cases when compared to non-cardiac deaths, revealing a drop in intracellular magnesium with myocardial ischemia [13,14]. Beyond cellular magnesium levels, environmental magnesium, particularly in drinking water, has been identified as an important factor in heart disease, where high magnesium levels (≥8.3 mg/L) were linked to reduced myocardial infarction mortality [13]. Conversely, platelet-activating factor (PAF) acts as a detrimental mediator in ischemic conditions. Animal studies in baboons, rats, sheep, and canines have demonstrated elevated PAF levels during ischemia and myocardial infarction, synthesized by cardiac endothelial cells and myocytes under hypoxic conditions [15]. Under this ischemic stress, ATP production is impaired, and shifts to an anaerobic metabolism. Consistent with these findings, PAF was found to exacerbate ischemic injury and impair cardiac function through thromboxane A2-mediated vasoconstriction and platelet activation [15]. In addition, PAF contributes to ischemia–reperfusion injury by promoting leukocyte adhesion and transmigration, which leads to myocardial necrosis; both effects decreased significantly with PAF receptor antagonists [15].

Angiogenesis (neoangiogenesis) is critical for tissue repair following myocardial infarction. Despite its pro-inflammatory effects, low levels of PAF, together with nitric oxide, have been shown to induce endothelial cell migration and the release of angiogenic factors, such as fibroblast growth factors [15]. Nevertheless, high levels of PAF can contribute to pathological angiogenesis through inflammation and angiogenesis can still occur in the presence of PAF receptor antagonists, indicating that PAF is not essential for this process [15].

Overall, both magnesium and PAF play critical yet opposing roles in ischemic heart disease and myocardial infarction, highlighting a valuable area for further research into their interconnected effects on cardiovascular health and shared, but opposing signaling pathways. While Magnesium has been shown to exert its cardioprotective function, as described, PAF differs through its sustained vasoconstrictive properties and impaired effect on cardiac myopathy recovery.

## 7. Cardiac Arrhythmias and Magnesium/PAF

Magnesium serves as an essential cofactor in cardiac electrophysiology, acting as a calcium antagonist and modulator of sodium, potassium, and ATP-dependent ion transporters. Beyond ischemic etiologies, disturbances in magnesium homeostasis have consistently been associated with heightened susceptibility to arrhythmias across a spectrum of clinical and experimental settings [13,16]. Hypomagnesemia is a well-established facilitator of both atrial and ventricular arrhythmias [4]. Its pro-arrhythmic effects are mediated by several mechanisms, including the promotion of ectopic beats, prolongation of cardiac repolarization, and a disruption of the resting membrane potential [17]. Epidemiological studies, such as the Framingham Heart Study, provide strong evidence for this association, linking low serum magnesium levels to an increased incidence of atrial fibrillation. Furthermore, the therapeutic role of magnesium is supported by evidence from randomized controlled trials and meta-analyses, which demonstrate that intravenous magnesium administration is effective in reducing the occurrence of postoperative and ischemia-related arrhythmias. Magnesium contributes to cardiac membrane stability by supporting ion pump function, limiting calcium influx, and regulating potassium currents, therefore preserving resting membrane potential and action potential duration. This stabilization reduces the likelihood of spontaneous depolarizations, indicating a clinically significant protective effect of sufficient magnesium levels on arrhythmia risk.

Emerging evidence suggests that magnesium deficiency may upregulate PAF-driven inflammatory pathways [17]. In vitro studies further substantiate this fact and demonstrate that magnesium suppresses platelet activation [15]. Elevated PAF concentrations have been observed in ischemic myocardium in the setting of magnesium deficiency [4]. Experimental data indicate that adequate magnesium may inhibit PAF biosynthesis, likely through stabilization of cellular membranes and attenuation of oxidative and cytokine-driven pathways [17]. Together, these findings raise the possibility that magnesium deficiency amplifies PAF-mediated inflammation and ionic dysregulation, ultimately promoting electrophysiologic instability. Prospective studies assessing magnesium status, circulating PAF, and electrophysiologic outcomes are needed to establish the ongoing knowledge gap that may definitively identify a magnesium-PAF mechanism responsible for metabolic and inflammatory dysregulation in arrhythmia pathogenesis, potentially revealing novel therapeutic strategies for both pathways.

## 8. Chronic Kidney Disease and Magnesium Role/PAF

Chronic Kidney Disease (CKD) is characterized by progressive disturbances in systemic electrolyte homeostasis, including alterations of circulating anions and cations that vary across disease progression. These disturbances contribute to broader systemic dysregulatory consequences which range from alterations in hemodynamic status to abnormalities in bone health [18].

Emerging evidence suggests PAF activity may be altered in CKD. Studies indicate that PAF acts as a renal inflammatory marker, promoting increased glomerular capillary permeability, leukocyte aggregation, and calcium dependent signaling within glomerular cells, leading to proteinuria and glomerulonephritis [19,20,21]. Clinical studies demonstrate increased urinary and plasma PAF levels, and increased plasma platelet aggravating factor acetylhydrolase (PAF-AH) activity in patients with primary glomerulonephritis compared to individuals with normal renal function [22].

These inflammatory processes contribute to progressive glomerular injury and may demonstrate an important link between dysregulated PAF activity and early development and progression of CKD. Patients in stages 3–4 of CKD have been reported to exhibit considerable increases in PAF-AH activity, the enzyme primarily responsible for PAF degradation [23]. Additional studies comparing chronic renal failure and CKD groups demonstrated correlations between increased PAF-AH activity and cardiac valve calcification [24]. Conversely, patients with arteriovenous fistulas (AVF) in end-stage renal disease exhibited higher serum PAF levels than healthy individuals [25]. While direct measurements of plasma PAF in CKD models are limited, increased PAF-AH activity may indicate a compensatory response to elevated PAF activity. Collectively, these findings suggest that PAF-AH could serve as a potential biomarker for platelet-mediated vascular dysfunction in CKD, and that alterations in the balance between PAF and PAF-AH levels may be associated with vascular involvement in disease progression.

Because the kidney plays a central role in electrolyte homeostasis, serum magnesium levels are commonly altered in CKD patients. Hypermagnesemia is correlated with advanced stages of CKD due to decreased GFR [19], while hypomagnesemia is correlated with earlier stages [17], and may contribute to CKD progression [20]. This relationship highlights the complex stage-dependent regulation of magnesium in CKD. A retrospective found both hypomagnesemia and hypermagnesemia were associated with increased mortality amongst CDK patients, although neither condition consistently correlated with CKD progression [26]. Furthermore, a recent small cohort study investigating oral magnesium supplementation and its effects on vascular calcification for CKD patients found no substantial difference in coronary artery calcification scores between control and experimental groups [24], emphasizing that further research is required to evaluate the vascular effect of different magnesium formulations on CKD stages. Given magnesium’s role as an inhibitor of platelet aggregation, magnesium deficiency may upregulate PAF in CKD patients and potentially exacerbate symptoms [20]. Clarifying the relationship between serum magnesium levels and PAF activity may provide further insight into the pathophysiology of CKD and may reveal novel treatments for therapeutic intervention. The majority of the findings on PAF Levels in primary glomerulonephritis suggest elevated levels in plasma and urine, urinary excretion, renal synthesis, serum acetylhydrolase activity, and glomerular damage. In summary, ongoing PGN switches PAF to a “hyperproduction” mode of within the kidney, resulting in higher urinary and plasma levels that correspond to compromised kidney function and heightened proteinuria [27].

## 9. Hemorrhaging and Magnesium Role

Magnesium and PAF are involved in studied mechanisms that contribute to hemorrhage in the body, most profoundly in intracranial hemorrhages [28]. As magnesium works to promote this vasodilating effect, the resulting drop in systemic resistance may expand hypovolemic effects due to the increased perfusion pressure. Interestingly, hypomagnesemia is observed in over 50% of subarachnoid hemorrhages [28]. Larger and more voluminous subarachnoid hemorrhages have produced even greater effects of hypomagnesemia, although the mechanism is still not clearly understood.

Magnesium is commonly found to have low serum levels in sites of hemorrhage, with more extensive hemorrhages associated with greater magnesium depletion [28]. The body may induce hypomagnesemia at sites of hemorrhage to prevent further anticoagulation. Consequently, hypomagnesemia could potentially be used as an indication for the presence of hemorrhage.

Although not traditionally classified as a hemorrhagic comorbidity, hypermagnesemia may compromise thrombus formation and exacerbate bleeding risk. This is further compounded when observing increased or higher magnesium levels in patients during bleeding times. A study showed a 48% reduction in platelet formation in healthy volunteers [23]. Given that hypermagnesemia is a potential comorbidity of hemorrhage, conditions known to induce hypermagnesemia—specifically CKD, hypothyroidism, and rhabdomyolysis—may be detrimental to the body’s physiological response to acute blood loss [18]. Age-related factors, including diminished renal clearance and increased use of magnesium-containing laxatives or antacids, further predispose elderly individuals to hypermagnesemia and its hemorrhagic sequelae [18].

## 10. Inflammation Pathway and PAF

**Figure 4:**
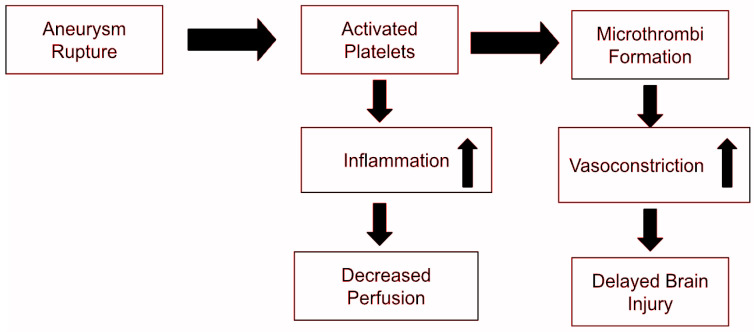
Modified by Kaine, A. Involvement of platelets in early brain injury and the pathogenesis of delayed cerebral ischemia. Following aneurysm rupture, erythrocytes and platelets are spilled into the subarachnoid space.

PAF is a potent phospholipid-derived mediator typically produced by endothelium and select leukocyte populations [6]. It plays a multifaceted role in hemostasis, enhancing vascular permeability, inducing vasospasm, and mediating leukocyte adhesion. However, excessive or dysregulated PAF can exert localized, yet systemic effects that exacerbate ischemic injuries. As illustrated in Figure 4, during ischemic conditions, PAF-activated leukocytes can amplify the inflammatory cascade, contributing to impaired perfusion [6]. The variable, mean arterial blood pressure (MAP), is a hallmark parameter of ischemic disease progression and is used as an indicator of prognostic findings. Importantly, given the role of inflammatory mediators within PAF mediated signaling pathways, PAF has been a useful biomarker within predicting several inflammatory-driven conditions. PAF, when synthesized and secreted by multiple cell types, is quickly hydrolyzed and degraded to an inactive metabolite, lyso-PAF, by the enzyme PAF acetylhydrolase [29].

Dysregulation of this pathway contributes to acute inflammatory conditions, such as pediatric anaphylaxis [29]. PAF has been shown to play a central role in allergic reactions, such as severe anaphylaxis, as well as other non-allergic-reaction-mediated diseases, including sepsis [29]. If these inflammatory disease states remain unresolved, chronic inflammatory conditions can come into fruition, linking carcinogenesis to chronic inflammatory disease processes. PAF signaling demonstrates this facilitated onset and progression of tumor growth through these inflammatory signaling pathways, leading to cancer etiologies [30]. Collectively, these findings highlight how PAF can serve both functionally as a clinically relevant biomarker of chronic inflammation and physiologically, when understanding the mechanistic link of PAF and severity of disease states or metastasis.

In PGN, levels of platelet-activating factor (PAF)—a pro-inflammatory mediator—are significantly elevated in both plasma and urine compared to healthy controls. These increased levels indirectly suggest excessive renal synthesis, which correlates with the disease activity and sustained proteinuria. In healthy human individuals, plasma platelet-activating factor (PAF) levels are maintained at very low concentrations to ensure homeostasis, typically measured around 54+40 pg/mL, although it can vary around 140 ± 122 pg/mL depending on the detection method. Because PAF is a potent mediator, it is rapidly degraded by the enzyme PAF acetylhydrolase (PAF-AH), keeping its concentration in check [30,31].

## 11. Hemorrhage and PAF

In subarachnoid hemorrhages, PAF levels exhibit a biphasic pattern characterized by an initial elevation, followed by a subsequent decline, attributed by an increased PAF-AH activity, an enzyme responsible for PAF degradation [32]. Certain diseases have exploited this enzymatic pathway to exacerbate hemorrhagic manifestations. For example, Leptospirosis, caused by *Leptospira interrogans*, has been shown to increase PAF-AH serum levels during pulmonary hemorrhage, reducing pulmonary PAF levels and impairing hemostatic functions [14]. Serum PAF-AH activity is thus utilized as a biomarker for monitoring disease progression of leptospirosis [14]. This also shows that PAF dysregulation could contribute further to hemorrhage risks associated with infectious diseases.

Studies have found PAF-induced vasospasm, although initially promoting hemorrhage control, may precipitate secondary ischemic infarction and even neurological deterioration if not alleviated [33]. This becomes problematic when hypercoagulable states persist, resulting in sustained vascular constriction and diminished or impaired cerebral perfusion. Observational data following subarachnoid hemorrhage have revealed that patients experiencing cerebral ischemia have elevated PAF levels, compared to those without ischemic complications [26]. Consequently, PAF antagonists are a rapidly rising area of research for promoting reperfusion during ischemia. As demonstrated in Figure 4, PAF-mediated vasospasm and thrombotic events in conjunction can exacerbate vascular dysfunction and can contribute to secondary ischemic responses [34].

Given the inverse physiological relationship between magnesium and PAF, their divergent trends during hemorrhage appear to reflect coordinated compensatory mechanisms aimed at regulating vascular tone and maintaining hemostatic equilibrium. Although a mechanistic interplay between Mg^2+^ and PAF remains unclear, we observe the body synergistically increasing PAF and decreasing serum Mg^2+^ levels to combat hemorrhage. Given magnesium’s vasodilatory and anti-aggregatory properties, it can be suggested that it may counteract the deleterious post-hemorrhagic effects of sustained PAF elevation, including ischemia and thrombosis. These factors, when in excess or in imbalance, also tend to exacerbate hemorrhage due to either promoting anticoagulation or increasing vascular permeability or thrombosis, leading to further hypovolemia and ischemia.

## 12. Magnesium Supplementation for Insomnia in Older Adults

There has been a recent upsurge in the use of Magnesium as an over-the-counter sleep aid for older adults with insomnia. Insomnia is characterized by prolonged sleep latency and is reported by around 50% of older adults over 55 years old [35]. Individuals with sleep insomnia have reported difficulties in falling asleep, or maintaining sleep, thus affecting daily function. Despite the increased use of magnesium for sleep regulation, the evidence supporting its efficacy and mechanism of action remains limited.

**Figure 5:**
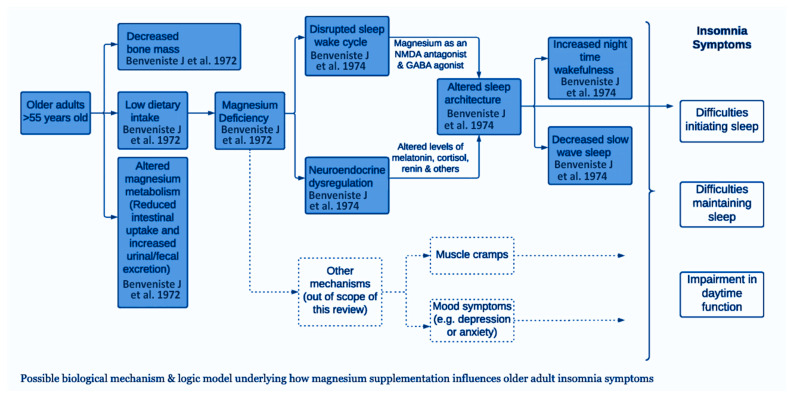
Framework of biological mechanisms leading to adult insomnia and magnesium influence as a supplementation [2,3].

As illustrated in Figure 5, a proposed framework outlines the biological mechanisms that may contribute to adult insomnia and the modulatory role of magnesium supplementation, including neuromodulated pathways (original pathway contributed by Jasmine Mah, PhD, and Tyler Pitre, MD, PhD). So far, it is known that older adults tend to have deficient magnesium levels leading to altered sleep through neuroendocrine dysregulation, such as altered melatonin, cortisol, and renin levels, all of which contribute to disturbed sleep in older adults [35]. On a neurochemical level, magnesium serves as an NMDA (N-methyl-D-aspartate) antagonist, which can disrupt normal synaptic transmissions leading to memory impairment and psychotomimetic effects [36]. Additionally, magnesium enhances γ-aminobutyric acid (GABA) signaling, which acts as an inhibitory neurotransmitter in the CNS causing neuronal hyperpolarization, thus decreasing neurotransmitter release [37]. Although there is data supporting the beneficial use of magnesium supplementation, current clinical research remains limited to fully explain its potential therapeutic effects, optimal dosing, and benefit of long-term use. Further investigation is warranted to justify its use in sleep pathophysiology, and overall efficacy in improving sleep quality and longevity in older adults.

## 13. Increased Platelet-Activating Factor in Sleep Apnea Patients with Hypoxemia

**Figure 6:**
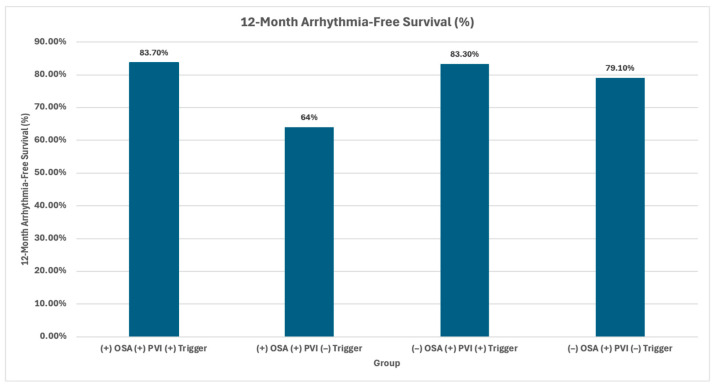
Modified by Karaolis, A. Kaplan–Meier survival curves according to treatment groups. OSA indicates obstructive sleep apnea, and PVI, pulmonary vein isolation. [Table contributed by Elad Anter, MD, and collaborators] Kaplan–Meier survival curves according to treatment groups. OSA indicates obstructive sleep apnea, and PVI, pulmonary vein isolation. [Table contributed by Elad Anter, MD, and collaborators].

Obstructive sleep apnea (OSA) has strong associations with increased risk of stroke, cardiovascular disease, hypertension, atherosclerosis, and vascular thrombosis. One of the prominent mechanisms linking obstructive sleep apnea (OSA) to cardiovascular disease is intermittent hypoxemia, which results from upper airway obstruction during sleep. These repeated reductions in oxygen concentration led to rapid drops in oxyhemoglobin levels, inducing oxidative stress, sympathetic activation, and the expression of prothrombotic factors, thus predisposing individuals to cardiovascular risks [33]. Figure 6 further illustrates the correlation between OSA and increased cardiovascular disease such as atrial fibrillation and incidence of arrhythmia.

**Figure 7:**
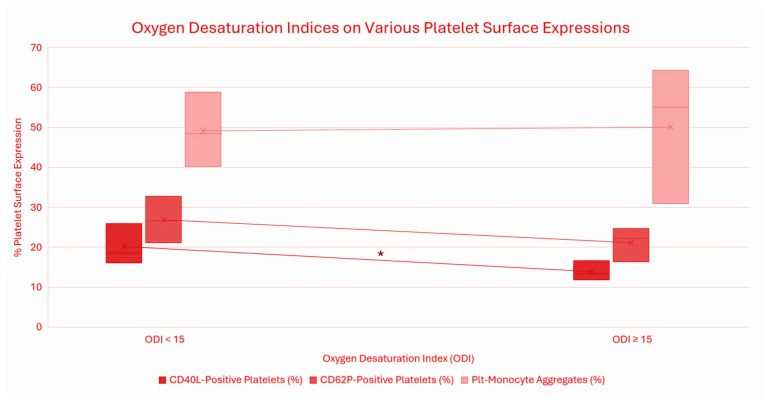
Modified box plot by Khan, E. Box plot with platelet surface expression values expressed as median [25th, 75th percentile]. Significant difference observed in CD40L-p0ositive platelet expression between mild hypoxemia (ODI < 15) and significant intermittent hypoxemia (ODI ≥ 15) (* *p* = 0.050). CD62P-positive platelet and Plt-monocyte aggregate expression showed no significant difference based on non-parametric Wilcoxon rank-sum test (Mann–Whitney U Test). All data and *p*-value calculations contributed by Dr. Ana Krieger, MD, and collaborators.

Recent studies have indicated a correlation between obstructive sleep apnea (OSA) and increased platelet activity (Figure 7). Platelet activation was compared in individuals with oxygen desaturation levels (ODI) greater or less than 15. The expression levels of platelet activating markers were used to show further implications between OSA and platelet activation. Markers such as CD40 ligand (CD40L) and P-selectin (CD62P) were expressed on the platelets’ surface, as well as levels of circulating platelet-monocytes [33]. Notably, CD40L expression was reduced on platelet surfaces in individuals with more severe cases of intermittent hypoxemia (ODI > 15), thus indicating an increase in platelet activation [33]. These findings suggest that OSA has an imposed risk of increased platelet mediated inflammation and prothrombotic factors, playing a key role in the development of cardiovascular complications. Therefore, further investigation into PAF and it signaling cascade may clarify its implications to disease progression, and therapeutic agents pertaining to its sleep-related vascular risks.

## 14. Magnesium-PAF and Type-2 Diabetes

Type 2 diabetic patients, particularly those with poor glycemic control and long disease duration, often present with hypomagnesemia. This deficiency is attributed to increased urinary magnesium excretion driven by both hyperglycemia and hyperinsulinemia. Type 2 diabetes is invariably associated with inflammation and platelet hyper reactivity; conditions linked to high levels of PAF. It is clear that diabetic complications flourish when tissue level of magnesium is low coupled with elevated PAF. The synergistic combination of hypomagnesemia and elevated PAF exacerbates vascular dysfunction, contributing to the progression of diabetic complications.

## 15. Discussion

Emerging evidence continues to underscore the indispensable role of magnesium as a critical electrolyte in maintaining physiological homeostasis and preventing disease progression. Magnesium exerts broad therapeutic effects through its inherent ability to regulate vascular tone, reduce inflammation, and inhibit platelet aggregation, functioning as a natural calcium antagonist [38]. Although calcium and magnesium often exert opposing physiological actions, their interplay maintains an essential biological equilibrium that can be quantified through the calcium-to-magnesium ratio. Dysregulation of this ratio has been linked to heightened cardiovascular risk, oxidative stress, endothelial dysfunction, and multiple organ pathologies [38].

Substantial literature supports the intricate relationship between magnesium (Mg^2+^) and platelet-activating factor (PAF) in modulating inflammatory and thrombotic processes. PAF contributes to platelet hyperactivity and propagates vascular inflammation via receptor-mediated intracellular calcium signaling. This elevation in intracellular calcium promotes macrophage activation, enhances low-density lipoprotein (LDL) oxidation, and accelerates atherogenesis. Conversely, magnesium attenuates this PAF-induced inflammatory cascade by suppressing calcium influx and stabilizing vascular and endothelial function [8].

In the context of myocardial infarction (MI), magnesium has demonstrated cardioprotective effects primarily through its role as a natural calcium channel blocker, reducing ischemic injury and improving myocardial performance. In contrast, PAF has been implicated in exacerbating ischemic damage via potent pro-thrombotic mechanisms. Hypomagnesemia further compounds these effects by elevating PAF levels during arrhythmic episodes, thereby amplifying platelet activation and vascular inflammation.

In chronic kidney disease (CKD), the magnesium–PAF axis exhibits a more complex interplay. Studies have reported concurrent elevations of PAF and its regulatory enzyme, PAF acetylhydrolase (PAF-AH), suggesting a compensatory feedback mechanism aimed at counterbalancing excessive PAF signaling in renal dysfunction. During ischemic and hemorrhagic events, both magnesium and PAF are central to the regulation of vascular inflammation and hemostatic balance. Moreover, magnesium’s role extends beyond the cardiovascular and renal systems into neurophysiological domains, including sleep regulation. Recent findings indicate that patients with obstructive sleep apnea demonstrate increased platelet activation, implicating PAF as a potential contributor to the cardiovascular comorbidities associated with sleep disorders.

## 16. Conclusions

Collectively, these findings highlight the multifaceted interaction between magnesium and PAF across cardiovascular, renal, neurological, and sleep-related pathologies. This relationship operates at multiple biological scales—from cellular and organellar signaling to systemic physiological regulation. Further experimental and clinical research is warranted to elucidate the precise molecular mechanisms underlying the magnesium–PAF interaction and to explore its therapeutic potential in modulating ischemic, hemorrhagic, and inflammatory disease pathways.

Importantly, dysregulation of magnesium homeostasis may potentiate this PAF-driven pathophysiology, contributing to the conditions described of ischemic injury, hemorrhagic conditions, hypocalcemia, diabetes, hypoxemia, inflammation and chronic kidney disease. Conversely, optimization of magnesium levels represents this therapeutic solution and a plausible counteracting strategy of opposing the effects of endothelial disruption and platelet aggregation. Overall, future studies can focus on defining tissue-specific effects of magnesium on PAF receptor signaling, as well as evaluating the efficacy of magnesium-based interventions in disease states driven by PAF mediated conditions and vasoconstrictive pathophysiology.

## Data Availability

No new data were created or analyzed in this study. Data sharing is not applicable to this article.
